# *Candida albicans* subverts host programmed cell death: a unified framework from pyroptosis to ferroptosis

**DOI:** 10.3389/fcimb.2026.1960578

**Published:** 2026-10-08

**Authors:** Limin Fan, Ruining Huang, Kexin Wang, Manting Ni, Xiuyu Nong, Zhenmou Xie, Bo Fu, Lijun Mo, Yinyuan Lan, Qingqing Xu, Wenhan Cao, Xinyue Li, Xueting Liu, Yaohua Xue

**Affiliations:** 1 Department of Laboratory, Dermatology Hospital, Southern Medical University, Guangzhou, China; 2 The Second Affiliated Hospital, The State Key Laboratory of Respiratory Disease, Guangdong Provincial Key Laboratory of Allergy & Clinical Immunology, Guangzhou Medical University, Guangzhou, China; 3 Nanfang Hospital, Southern Medical University, Guangzhou, China; 4 Department of Pathology, The Third Affiliated Hospital of Southern Medical University, Guangzhou, China

**Keywords:** *Candida albicans*, candidiasis, cell death, host immune response, inflammatory cells, virulence factors

## Abstract

*Candida albicans* is a major opportunistic fungal pathogen that causes substantial morbidity and mortality, particularly in immunocompromised individuals. Increasing evidence indicates that its pathogenicity extends beyond classical virulence traits and involves active engagement of host programmed cell death (PCD) pathways. In this review, we propose a unified mechanistic framework to explain how *C. albicans* virulence programs—including hyphal morphogenesis, candidalysin-mediated epithelial injury, cell wall remodeling, and metabolic adaptation—interact with host sensing systems to shape distinct yet interconnected forms of cell death. Among the currently implicated pathways, pyroptosis is the most strongly supported and represents the clearest link between fungal invasion, inflammasome activation, and inflammatory tissue injury. By contrast, apoptosis appears to be more heterogeneous and context-dependent, whereas necroptosis and ferroptosis remain emerging mechanisms whose contributions likely vary across cell types, tissues, and stages of infection. We further examine how host compartments, including epithelial cells, macrophages, neutrophils, and IL-17-associated immune circuits, amplify, buffer, or redirect these death responses, thereby influencing the balance between fungal clearance and immunopathology. Particular emphasis is placed on tissue specificity, as the biological consequences of PCD differ markedly across oral, vaginal, intestinal, hepatic, and disseminated candidiasis. Finally, we discuss the translational implications of this framework for host-directed antifungal strategies and argue that therapeutic modulation of cell death will require careful attention to timing, tissue context, and preservation of protective immunity. This review provides an evidence-weighted and integrative perspective on how *C. albicans* subverts host cell death and how these processes may be leveraged for future therapeutic innovation.

## 1 Introduction

*Candida albicans* is a commensal fungus of the human mucosa and an opportunistic pathogen capable of causing a broad spectrum of diseases, from superficial mucosal infections to life-threatening disseminated candidiasis (Nouraei et al., 2020). Its pathogenicity does not rely solely on fungal burden, but rather on a dynamic interplay among fungal virulence programs (Ponde et al., 2021), host immune sensing, and tissue-specific responses (Valand and Girija, 2021). Over the past decade, substantial progress has been made in defining the major virulence determinants of *C. albicans*, including morphological plasticity (Cota and Hoyer, 2015; Ahmed et al., 2026; Yang et al., 2026), cell wall remodeling (Tsai et al., 2026), metabolic adaptation, adhesion mediated by the ALS family (Hoyer et al., 2024), and the secretion of damaging effectors such as candidalysin. Concurrently, studies of antifungal immunity have revealed the complex roles of epithelial barriers (Möslinger et al., 2026), myeloid cells, and IL-17-associated pathways in shaping both fungal control and inflammatory pathology (Ferreira-Gomes et al., 2021; Martini et al., 2026).

In parallel to these advances, programmed cell death (PCD) has emerged as a central mechanism linking fungal recognition to tissue outcome. Initially regarded primarily as a terminal consequence of infection, PCD is now understood to function as a highly regulated immunological process that can either enhance host defense or amplify disease. In *C. albicans* infection, inflammatory and non-inflammatory death pathways—including pyroptosis, necroptosis, apoptosis, and ferroptosis—may influence fungal clearance, cytokine release, barrier integrity, and organ injury (Chen et al., 2024; Jiang et al., 2024; Sze et al., 2026). However, these pathways are often discussed in isolation, whereas *in vivo* they are embedded within a broader network shaped by fungal morphogenesis, host cell type, local metabolic conditions, and inflammatory feedback loops (Weerasinghe et al., 2026).

Given this complexity, a major unresolved question is not simply whether *C. albicans* induces host cell death, but how specific fungal traits are translated into distinct death programs in different cellular and tissue contexts. To address this question, we organize this review around a unified framework in which *C. albicans* virulence factors act as triggers, host immune and stromal compartments function as amplifiers or modulators, and PCD pathways serve as the execution layer that determines whether the net outcome is protective fungal control or damaging immunopathology. This framework is especially useful for understanding why similar fungal signals can produce markedly different consequences in oral, vaginal, gastrointestinal, hepatic, or systemic candidiasis.

Importantly, the evidence supporting individual death pathways is not equivalent. Pyroptosis is relatively well established as a host response to *C. albicans*, particularly in macrophages and inflamed barrier tissues. By contrast, evidence for necroptosis and ferroptosis remains more limited and context-dependent, while other pathways such as cuproptosis remain speculative in candidiasis and are best considered future directions rather than established mechanisms. We therefore distinguish throughout this review between well-supported mechanisms, emerging but incompletely validated pathways, and hypothesis-generating concepts.

Rather than providing a broad catalog of all immune cell subsets or all reported disease associations, we focus specifically on the mechanistic interfaces among fungal virulence, host immune amplification, and cell death regulation. Our aim is to identify the key molecular and cellular “switches” that determine when host death responses are beneficial, when they become pathological, and how this knowledge may inform host-directed antifungal strategies without overlooking the risk of impairing fungal clearance.

## 2 *Candida albicans* virulence factors as upstream triggers of host cell death

To understand how programmed cell death shapes the outcome of candidiasis, it is first necessary to consider the fungal traits that initiate host damage and danger signaling. We therefore begin by examining the major *Candida* virulence factors that act as upstream triggers of host cell stress and death-associated responses.

The relationship between *Candida* infection and host cell death begins with the pathogen itself. Rather than being passive consequences of fungal presence, many death-associated host responses are initiated by specific virulence programs that enable invasion, damage, and immune activation. The ability of *C. albicans* to induce or reshape host cell death does not arise from a single toxin or signaling event. Instead, it reflects the coordinated action of multiple virulence determinants that generate mechanical stress, membrane damage, metabolic competition, and innate immune activation. In this context, fungal virulence factors should not be viewed merely as markers of pathogenicity; they function as upstream death-shaping signals that establish whether host cells remain viable, activate inflammatory programs, or undergo regulated death. Importantly, these fungal determinants rarely act alone. Their effects are integrated by host receptors, inflammatory mediators, and tissue-specific constraints, which together determine the dominant PCD phenotype.

### 2.1 Morphological transition: a mechanical and immunological trigger of inflammatory cell death

Among the best-characterized virulence traits of *C. albicans* is its capacity to transition between yeast, pseudohyphal, and hyphal forms. This morphological plasticity is essential for tissue invasion, immune evasion, and dissemination, but it also has direct implications for host cell death. In macrophages, the yeast-to-hypha transition is more than a correlate of virulence: it is a major trigger of intracellular stress. Hyphal expansion within the phagosome imposes physical strain on host membranes, alters phagosomal integrity, and promotes conditions that favor inflammasome activation and pyroptotic signaling (Westman et al., 2018; Ding et al., 2021).

This morphological trigger is particularly relevant because it links fungal growth behavior to a defined host death response (Olivier et al., 2022). Hypha-associated stress can cooperate with innate immune priming signals to activate inflammatory caspases and gasdermin-dependent membrane permeabilization (Zhou et al., 2025). As a result, the transition to filamentous growth may convert a successfully phagocytosed fungal cell into a stimulus for lytic death of the host phagocyte. At the same time, this process is not uniformly detrimental to the host. Pyroptotic death of infected macrophages can enhance alarm signaling and cytokine release, but it may also facilitate fungal escape from the intracellular niche and reduce the capacity of phagocytes to contain infection (Wellington et al., 2014; Weerasinghe et al., 2026).

Thus, the morphogenetic program of *C. albicans* should be understood as a central upstream driver of the “protective versus pathological” balance in candidiasis: the same hyphal transition that promotes fungal invasion can also trigger host inflammatory defenses, yet when excessive or poorly controlled, it may shift the response toward tissue-damaging cell death (Shi et al., 2009; Luan et al., 2020; Wu et al., 2021).

### 2.2 Candidalysin: toxin-mediated epithelial damage and inflammatory amplification

A second major determinant connecting fungal virulence to host cell death is candidalysin, the peptide toxin secreted by hyphal *C. albicans* (Moyes et al., 2016). Candidalysin disrupts epithelial membrane integrity and activates danger-associated signaling pathways, thereby serving as both a direct damage factor and a potent amplifier of local inflammation (Konwar et al., 2025). Its significance lies not only in its cytolytic effects, but also in its ability to transform epithelial cells from passive barrier targets into active participants in antifungal signaling (Verma et al., 2017; Richardson et al., 2018; Chu et al., 2020; Konwar et al., 2025).

Exposure to candidalysin can induce membrane perturbation, calcium flux, MAPK activation, alarmin release, and cytokine production (Wang et al., 2021), thereby creating a tissue environment permissive for inflammatory amplification and, in some settings, programmed cell death (Ho et al., 2020; Ponde et al., 2022; Lin et al., 2024; Thapa et al., 2025). In mucosal tissues, candidalysin-driven injury may act synergistically with fungal burden and host cytokine networks to lower the threshold for inflammasome activation or other death-associated stress responses. However, the consequences are highly cell-type dependent. In epithelial compartments, candidalysin prominently contributes to barrier damage and inflammatory recruitment, whereas in professional immune cells its role may be more indirect, acting through priming signals, ion imbalance, or damaged-tissue mediators rather than serving as a universal direct inducer of death (Kasper et al., 2018; Konig et al., 2020).

This distinction is important for interpretation of the literature (Paulin et al., 2026). Candidalysin is clearly a major pathogenic determinant, but its relationship to specific PCD pathways should be defined in a tissue- and cell-specific manner rather than generalized across all forms of candidiasis (Namvar et al., 2026).

### 2.3 Cell wall remodeling and pattern exposure: licensing host death responses

The fungal cell wall is the primary interface between *C. albicans* and host innate immune sensors. Dynamic changes in the exposure of β-glucan, mannan, and chitin influence recognition by receptors such as Dectin-1 and related pattern-recognition systems, thereby shaping the inflammatory context in which programmed cell death occurs. These interactions do not necessarily trigger host death directly; instead, they often function as licensing signals that prime or potentiate downstream death pathways (Namvar et al., 2026).

For example, β-glucan recognition can enhance inflammatory transcriptional programs, promote cytokine production, and lower the activation threshold for inflammasome-associated responses (Wu et al., 2020; Kumar et al., 2022; Valand et al., 2022). In this way, cell wall remodeling links fungal adaptation to host susceptibility for inflammatory cell death. At the same time, the consequences of wall exposure are not fixed. Depending on fungal morphology, burden, and host compartment, the same pattern-recognition events may support protective antifungal immunity or feed into excessive inflammatory activation (Villar et al., 2012).

Accordingly, cell wall pathogen-associated molecular patterns (PAMPs) are best viewed not as isolated immunological signals, but as part of a broader network through which *C. albicans* configures the host death landscape.

### 2.4 Metabolic adaptation and nutrient competition: stress pathways beyond classical pattern recognition

In addition to structural and toxin-mediated virulence factors, *C. albicans* shapes host responses through metabolic adaptation and competition for nutrients within infected niches. During phagocytosis or tissue invasion, fungal growth can alter local availability of glucose, amino acids, iron, oxygen, and other metabolites, thereby creating stress conditions that influence host survival and death decisions (Tada et al., 2002; Tucey et al., 2020; Li et al., 2025). These effects are particularly relevant in phagocytes and inflamed mucosal tissues, where metabolic limitation and reactive oxygen species may cooperate with inflammatory signaling (Sener et al., 2007; Inoue et al., 2009).

Such metabolic competition may help explain why host cell death in candidiasis cannot be attributed solely to receptor-mediated recognition (Cao et al., 2019; Tucey et al., 2020; Zhang et al., 2024). Instead, fungal infection often imposes a composite stress state involving bioenergetic pressure, oxidative imbalance, lysosomal dysfunction, and inflammatory priming (Millet et al., 2022; He et al., 2024). These converging signals may favor pyroptosis in some contexts, while potentially contributing to other regulated death modalities, including ferroptosis-related lipid peroxidation or compensatory death responses when canonical inflammasome pathways are insufficient (Zhao et al., 2024).

Although many of these mechanisms remain incompletely resolved, they underscore a central principle of this review: fungal virulence factors influence host cell death not only through direct damage, but also by remodeling the metabolic and inflammatory conditions under which death pathways are activated.

### 2.5 Summary: from virulence traits to death-inducing conditions

Taken together, *C. albicans* virulence factors do not map one-to-one onto individual host death pathways. Rather, they generate a set of overlapping conditions—mechanical stress, membrane injury, inflammatory priming, and metabolic disruption—that make specific forms of host cell death more or less likely. This perspective helps explain why the same fungus can trigger distinct outcomes across tissues and why virulence and host death should be analyzed as an integrated system rather than as separate topics.

*Candida albicans* virulence programs, including hyphal morphogenesis, candidalysin secretion, cell wall remodeling, and metabolic adaptation, generate overlapping forms of host stress such as membrane injury, inflammasome activation, oxidative stress, and barrier disruption. These components play the roles of amplifier, buffer and switch in the cell death response. The resulting programmed cell death pathways—most prominently pyroptosis, but also apoptosis, necroptosis, ferroptosis, and potentially PANoptosis—can either support fungal containment or drive tissue injury, depending on the tissue context, timing, and magnitude of activation. We have summarized the information in **Figure 1**.

**Figure 1:**
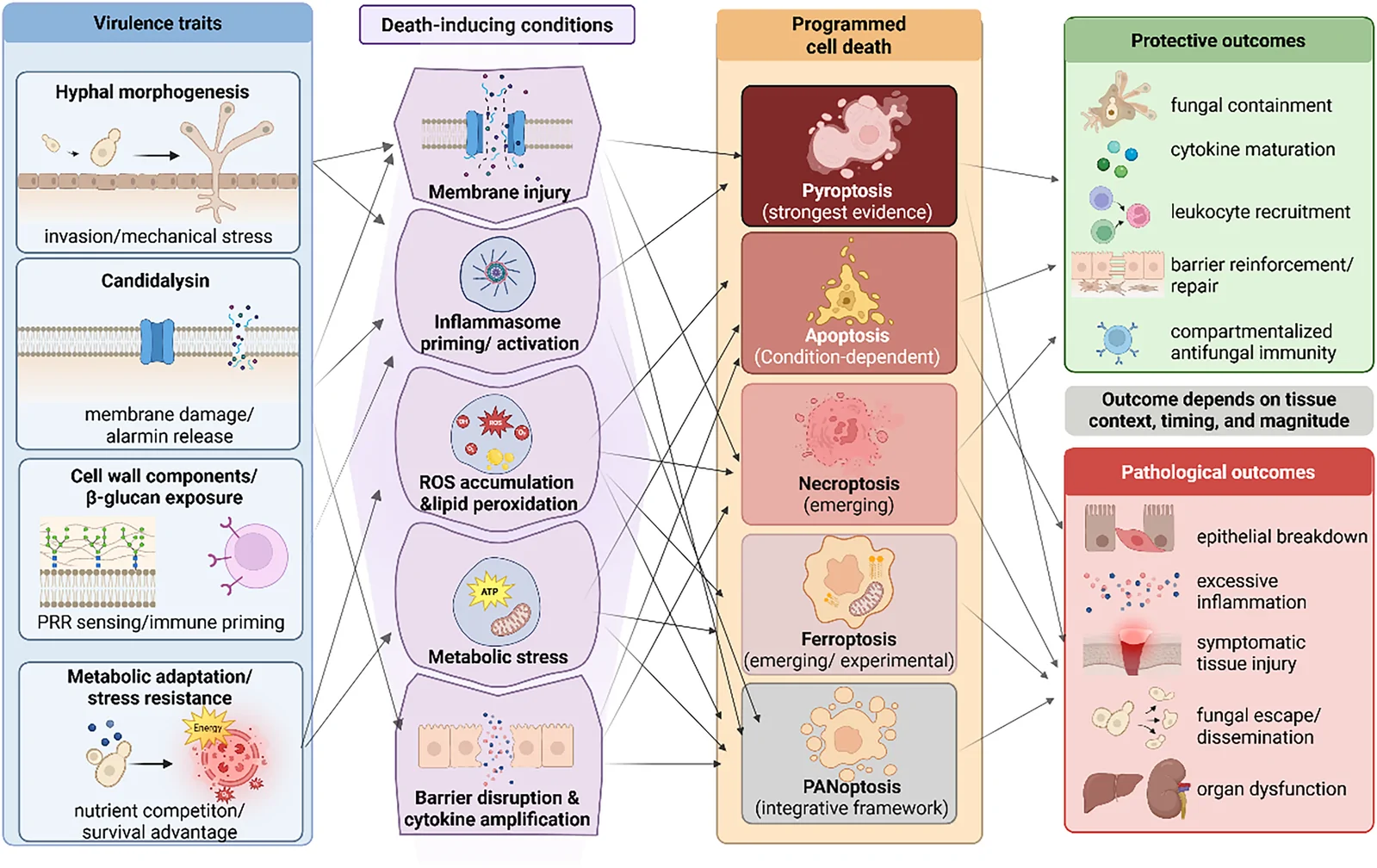
A unified framework linking *Candida albicans* virulence traits, host compartments, and programmed cell death outcomes.

## 3 Host immune and stromal compartments as amplifiers of candida-induced cell death

Once fungal virulence traits engage the host, the next critical determinant is how these signals are detected, amplified, and interpreted by different cell types. Thus, the biological consequences of Candida-induced damage depend not only on the fungal trigger, but also on the host compartments that convert that trigger into inflammation and cell death.

Host cells are not merely passive victims of fungal injury. Different immune and stromal compartments interpret, amplify, restrain, or redirect *C. albicans*-derived stress signals in ways that profoundly affect the form and consequences of programmed cell death. For this reason, the relevant question is not only which fungal factors are present, but also which host cells receive the signal, in what sequence, and within what inflammatory milieu. To maintain a mechanistic focus, we discuss here only those cellular compartments most directly involved in shaping cell death outcomes during candidiasis.

### 3.1 Macrophages: the best-defined platform for candida-induced pyroptosis

Among host cell types, macrophages provide the clearest example of how *C. albicans* virulence intersects with programmed cell death. Following phagocytosis, macrophages integrate fungal morphology, phagosomal integrity, nutrient stress, and innate immune priming to determine whether engulfed fungi are controlled or instead trigger inflammatory cell death, and host factors such as Trim72 can protect against lethal *C. albicans* infection (Tan et al., 2024). A substantial body of work supports a central role for inflammasome activation and pyroptosis in this context, particularly involving NLRP3, caspase-1, and gasdermin-dependent membrane permeabilization (Wellington et al., 2014; Kasper et al., 2018; Ding et al., 2021).

Macrophage pyroptosis is functionally ambivalent. On one hand, it promotes the release of IL-1β and IL-18, enhances inflammatory recruitment, and may support antifungal Th1/Th17 responses (van de Veerdonk et al., 2011). On the other hand, premature lytic death can compromise intracellular fungal containment and allow hyphal escape (Ding et al., 2021; Olivier et al., 2022), thereby converting an initially protective response into a mechanism of fungal persistence or tissue injury. This duality makes macrophages a central “decision node” in candidiasis: they do not simply execute death but determine whether death strengthens host defense or undermines it.

Macrophages are also important because they illustrate how multiple upstream fungal signals converge on a shared outcome. Hyphal morphogenesis, candidalysin-associated damage signals, cell wall–driven priming, and metabolic competition can all influence macrophage death thresholds (Tucey et al., 2020; Ding et al., 2021). As such, they represent the most experimentally grounded model for linking fungal virulence, host sensing, and PCD execution.

### 3.2 Neutrophils: antifungal effectors that can also intensify collateral injury

Neutrophils are indispensable for host defense against invasive candidiasis, particularly through phagocytosis, degranulation, reactive oxygen species production, and neutrophil extracellular trap (NET) formation (Urban et al., 2006; Sakuma et al., 2025). However, impaired NET-forming capacity can contribute to susceptibility to chronic vaginitis in vulvovaginal candidiasis (VVC) models (Yano and Fidel, 2024). The molecular mechanisms of neutrophil regulatory networks in anti-Candida infection have been increasingly defined (Xu et al., 2025). However, their contribution to the cell-death landscape is more complex than direct fungal killing alone. By releasing proteases, oxidants, and inflammatory mediators, neutrophils can shape the survival of neighboring epithelial, endothelial, and myeloid cells and thereby influence whether tissue responses remain protective or become destructive.

Compared with macrophages, the direct mechanistic coupling between neutrophils and specific PCD pathways in candidiasis is less well defined. Nevertheless, neutrophil-driven oxidative stress may create conditions permissive for secondary injury and possibly for death programs linked to lipid peroxidation or membrane damage in surrounding tissues (Millet et al., 2022; Zhao et al., 2024). In mucosal disease, neutrophil influx may be prominent even when fungal clearance is incomplete (Lionakis et al., 2012; Lionakis et al., 2017; Yano et al., 2018), highlighting the possibility that inflammatory amplification can become partly uncoupled from effective host protection.

Thus, neutrophils should be viewed as major modulators of tissue outcome rather than simply as terminal effectors of antifungal immunity.

### 3.3 Epithelial cells: barrier sentinels and local executors of inflammatory injury

Epithelial cells occupy a pivotal position in candidiasis because they are both the first line of defense and a major site of fungal-induced damage (Mills et al., 2024). They sense fungal morphology, respond to candidalysin, produce chemotactic and inflammatory mediators, and determine whether barrier perturbation remains contained or escalates into broader immunopathology. In this sense, epithelial cells are not passive substrates of virulence; they actively translate fungal invasion into tissue-level inflammatory programs.

Recent studies suggest that epithelial injury may also intersect with inflammatory cell death pathways, particularly in mucosal candidiasis. Epithelial pyroptosis, for example, has been proposed to contribute to macrophage recruitment and inflammatory amplification in oral infection (d'Enfert et al., 2021; Pellon et al., 2026). Such observations are important because they expand the PCD framework beyond phagocytes and indicate that tissue-resident structural cells can shape the inflammatory trajectory of disease.

The epithelial compartment therefore acts as both a barrier and a signaling hub, integrating fungal toxins, microbial burden, and immune-derived cytokines to determine whether local death responses serve host protection or drive symptomatic tissue damage.

### 3.4 IL-17-associated circuits: protective barrier immunity with context-dependent inflammatory costs

The IL-17 axis is a central component of antifungal immunity at mucosal surfaces (Conti et al., 2009; Gaffen et al., 2011; Gladiator et al., 2013), where it promotes epithelial defense, antimicrobial peptide production, and recruitment of neutrophil-associated responses (Aggor et al., 2024).In addition to Th17 cells, innate lymphoid cells can integrate fungal sensing and plasticity to control fungal infections and contribute to type 17 immunity (Tsymala et al., 2026). IL-17-mediated antifungal defense can operate even independently of classical fungal innate immune receptors in epidermal clearance of *C. albicans* (Iwasawa et al., 2022). In most settings, these functions are protective and are crucial for resistance to mucocutaneous candidiasis, although IL-17-mediated antifungal defense in the oral mucosa can be independent of neutrophils (Trautwein-Weidner et al., 2015). Optimal IL-17-mediated immunity to oral mucosal candidiasis also requires epithelial IL-22/STAT3 signaling (Aggor et al., 2020). Human genetic evidence further supports this axis, as gain-of-function STAT1 mutations impair IL-17 immunity and underlie chronic mucocutaneous candidiasis (Liu et al., 2011). However, IL-17-driven inflammation does not operate independently of tissue damage and cell-death pathways. When epithelial injury, inflammasome activation, or excessive leukocyte recruitment is already established, IL-17-associated responses may contribute to a feed-forward inflammatory circuit rather than purely protective containment. Beyond classical immune cells, oral megakaryocytes activated by IL-17 and candidalysin can also mediate immunity to pathogenic mucosal *C. albicans* infections (Launder et al., 2024).

For this reason, IL-17 signaling should not be treated in this review as a separate immunological mini-topic. Instead, it is best understood as a context-dependent modifier of the cell-death environment—one that may reinforce barrier defense under controlled conditions yet also intensify immunopathology when tissue injury and inflammatory death are already amplified.

### 3.5 Summary: host cells as amplifiers, buffers, and switches

Collectively, macrophages, neutrophils, epithelial cells, and IL-17-associated circuits do not merely respond to *C. albicans*; they shape the form, intensity, and consequence of programmed cell death. This helps explain why similar fungal stimuli can produce divergent outcomes across tissues. In some settings, host cell death contributes to containment and immune coordination. In others, the same processes amplify barrier failure, inflammatory recruitment, and organ damage. Understanding these host compartments as amplifiers and switches, rather than as isolated immune actors, provides the conceptual bridge to the pathway-specific discussion in the next section, consistent with the context-dependent redundancy and specificity of chemokine receptors (Dyer et al., 2019).

## 4 Programmed cell death pathways in candidiasis

Programmed cell death represents the point at which upstream fungal signals and host inflammatory circuits are translated into concrete cellular outcomes. In candidiasis, multiple death pathways have been proposed, but the strength of evidence and mechanistic resolution vary substantially among them. Programmed cell death in candidiasis should not be viewed as a collection of parallel, independent pathways. Rather, different death programs represent context-dependent outcomes of a shared host–pathogen interaction network shaped by fungal morphogenesis, toxin-mediated injury, innate immune priming, metabolic stress, and tissue environment. Among the currently discussed pathways, pyroptosis is the most firmly supported mechanism in *Candida albicans* infection, particularly in macrophages and inflamed mucosal settings. By contrast, apoptosis, necroptosis, and ferroptosis are better considered as contextual or emerging components whose contribution may vary by cell type, organ, and disease severity. More integrative concepts such as PANoptosis may help explain overlapping death phenotypes, but their mechanistic definition in candidiasis remains incomplete.

For clarity, we discuss these pathways separately below while recognizing that, *in vivo*, their activation is often overlapping, sequential, or compensatory rather than strictly isolated.

### 4.1 Pyroptosis: the best-established inflammatory death pathway in candidiasis

Pyroptosis is currently the most extensively characterized programmed cell death pathway in *C. albicans* infection. It is a lytic and strongly pro-inflammatory form of cell death typically associated with inflammasome activation, caspase-1 signaling, gasdermin cleavage, membrane pore formation, and the release of IL-1β and IL-18 (Shi et al., 2015). In candidiasis, pyroptosis is particularly well supported in macrophages, where fungal uptake is followed by a sequence of events involving innate immune priming, phagosomal stress, hyphal expansion, and inflammasome activation.

A key reason pyroptosis occupies a central position in candidiasis is that it provides a mechanistic link between major fungal virulence traits and host inflammatory outcome. Hyphal morphogenesis can disrupt phagosomal homeostasis and increase membrane stress (Vylkova and Lorenz, 2017; Shi et al., 2025), while cell wall exposure and danger signaling provide permissive conditions for inflammasome activation (Rogiers et al., 2019; Zhou et al., 2025). In this setting, pyroptosis is not merely a downstream consequence of overwhelming infection; it is part of the host’s active response to intracellular fungal challenge. This helps explain why *C. albicans*, unlike many less invasive commensal fungi, is particularly effective at eliciting inflammatory death responses in myeloid cells.

The functional consequence of pyroptosis, however, is distinctly double-edged. On one hand, pyroptosis promotes cytokine maturation, inflammatory recruitment, and local amplification of antifungal immunity. IL-1 family signaling can support protective leukocyte responses and contribute to the development of downstream adaptive immunity, including Th17-associated antifungal defense (van de Veerdonk et al., 2011). On the other hand, pyroptotic lysis destroys infected phagocytes, may permit fungal escape (Weerasinghe et al., 2026), and can intensify tissue inflammation beyond what is required for fungal control. Thus, pyroptosis should not be categorized as simply beneficial or harmful; its impact depends on timing, magnitude, and tissue context.

This balance is particularly relevant in mucosal candidiasis. Recent evidence suggests that pyroptosis-related signaling may also occur beyond macrophages, including in epithelial-rich inflammatory environments where local cell death contributes to recruitment and symptomatic tissue injury (Lian et al., 2022; Peng et al., 2024; Shang et al., 2025). In these settings, pyroptosis may help initiate antifungal defense but also participate in barrier disruption and feed-forward inflammation. Such findings broaden the relevance of pyroptosis from a macrophage-centered mechanism to a more general inflammatory logic in candidiasis, while still recognizing that the strongest mechanistic evidence remains in myeloid cells.

Taken together, available data support pyroptosis as the reference pathway for understanding Candida-induced inflammatory cell death: it is the best-established example of how fungal virulence, innate immune sensing, and tissue pathology converge. This makes pyroptosis the most immediate candidate for mechanistically informed host-directed intervention, although any attempt to suppress it therapeutically must account for the possibility of impaired fungal containment.

### 4.2 Apoptosis: a non-lytic pathway with context-dependent roles in immune regulation and tissue injury

Compared with pyroptosis, apoptosis has received less attention as a defining feature of candidiasis, yet it remains relevant because it represents a fundamentally different host outcome. Unlike pyroptosis, apoptosis is generally non-lytic and less inflammatory, allowing infected or stressed cells to be removed with more limited release of intracellular danger signals (Blagojevic et al., 2021). In principle, this may favor resolution and reduce collateral tissue injury. However, in the context of infection, apoptosis can also deplete protective immune cells or weaken barrier integrity if it occurs excessively (Chen et al., 2024; Jiang et al., 2024).

During *C. albicans* infection, apoptotic responses have been reported in several cellular settings, including epithelial (Wagener et al., 2012) and immune compartments (Villar et al., 2012), but the mechanisms are often heterogeneous and less clearly linked to a single canonical trigger than is the case for inflammasome-driven pyroptosis. Oxidative stress, mitochondrial dysfunction, cytokine imbalance, and prolonged inflammatory activation may all contribute (Sze et al., 2026). In some contexts, apoptosis may represent a secondary or compensatory response when lytic inflammatory pathways are not dominant; in others, it may coexist with pyroptotic or necrotic features (Naik and Mohammed, 2023).

The importance of apoptosis in candidiasis therefore lies less in its role as a uniquely defining pathway and more in its contribution to the overall balance between controlled cell turnover and inflammatory destruction. If apoptotic clearance is efficient, tissue homeostasis may be preserved. If apoptotic programs become extensive or are accompanied by defective clearance, they may instead contribute to barrier compromise, immune suppression, or transition to more damaging forms of death.

Accordingly, apoptosis should be considered a relevant but not central framework for *C. albicans*-induced pathology (Roman-Carraro and Gutierrez-Kobeh, 2026). It provides an important comparator to pyroptosis by illustrating that not all infection-associated host cell death is inflammatory, and that the mode of death can substantially influence downstream immune consequences.

### 4.3 Necroptosis: a plausible but incompletely defined contributor to candida-associated injury

Necroptosis is a regulated lytic cell death pathway classically associated with RIPK1, RIPK3, and MLKL signaling. Because it shares membrane-disruptive and pro-inflammatory features with pyroptosis, necroptosis has attracted interest as a potential contributor to tissue damage in fungal infection (Cao et al., 2019). However, in candidiasis, the current evidence base is substantially less mature than that for pyroptosis.

Existing studies suggest that necroptosis-associated signaling can drive inflammatory injury under certain conditions. TSC1 inhibits necroptosis in macrophages to constrain *Candida albicans* infection, an outcome that becomes particularly relevant when host tissues encounter severe stress, cytokine-rich microenvironments, or crosstalk with alternative cell death pathways (Li et al., 2021). It is biologically plausible that *C. albicans*-induced membrane damage, oxidative stress, and inflammatory amplification could create conditions favorable for necroptotic responses in some cell types (Lian et al., 2022; Wang et al., 2023). Nevertheless, direct pathway-resolved evidence in candidiasis remains limited, and it is often difficult to distinguish bona fide necroptosis from mixed lytic death phenotypes driven by inflammasomes, secondary necrosis, or generalized tissue destruction (Banoth et al., 2020).

For this reason, necroptosis is best framed at present as an emerging mechanism rather than an established core pathway in *C. albicans* disease. It may prove most relevant in organ injury contexts where inflammatory damage exceeds localized fungal control, but stronger evidence—particularly based on pathway-specific genetic or pharmacologic interrogation in defined models—is still needed.

This distinction is important both conceptually and therapeutically. Overstating necroptosis risks blurring the stronger mechanistic case for pyroptosis, whereas underappreciating it may obscure potentially important tissue-specific injury pathways. A balanced view is therefore to recognize necroptosis as biologically credible, experimentally suggestive, but not yet a dominant explanatory model for candidiasis.

### 4.4 Ferroptosis: an emerging metabolic-death hypothesis in inflammatory candidiasis

Ferroptosis is an iron-dependent form of regulated cell death characterized by lipid peroxidation, oxidative membrane damage, and failure of cellular antioxidant defenses (Zhou et al., 2024). Because *C. albicans* infection can alter iron availability, reactive oxygen species production, and inflammatory metabolism, ferroptosis has emerged as a conceptually attractive pathway in candidiasis. It may be particularly relevant in tissues where oxidative stress, myeloid infiltration, and metabolic disruption are pronounced (Xiao et al., 2025). Ferroptosis has also been implicated in organ remodeling and fibrosis in impaired organs (Yin et al., 2024).

Recent studies have begun to suggest that ferroptosis-related processes may contribute to pathological inflammation in selected forms of candidiasis, including oral (Li et al., 2026), vagina (Hou et al., 2026) and systemic settings (Millet et al., 2022; Wei et al., 2026). These observations are notable because they expand the discussion of fungal pathogenesis beyond classical receptor-mediated immunity and highlight how infection-driven metabolic stress might itself become a determinant of tissue injury. In this view, ferroptosis would not simply be another death pathway added to the list, but a mechanism linking inflammatory burden, lipid damage, and organ dysfunction.

At the same time, compared with ferroptosis, the evidence is still preliminary. The evidence for iron death mediated by EphA2/IL-23 and CVF1 during candidiasis comes from *in vitro* studies and animal models (Millet et al., 2022; Luo et al., 2025). Moreover, oxidative injury during candidiasis may reflect overlapping processes, including neutrophil-derived reactive species, mitochondrial dysfunction, and nonspecific inflammatory damage. Therefore, although ferroptosis is an important and promising area of investigation, it should currently be regarded as an emerging, context-dependent mechanism rather than a uniformly established feature of *C. albicans* infection (Bao et al., 2024).

Even with these limitations, the ferroptosis framework is useful because it broadens how we think about host damage in candidiasis. It suggests that fungal pathogenesis may be shaped not only by immune recognition and toxin injury, but also by infection-induced shifts in iron handling, redox balance, and lipid vulnerability.

### 4.5 PANoptosis: a useful integrative concept, but not yet a fully defined mechanism in candidiasis

PANoptosis has been proposed as an integrated inflammatory cell death framework that incorporates features of pyroptosis, apoptosis, and necroptosis within a coordinated signaling platform rather than treating these pathways as mutually exclusive. This concept has gained traction in infectious and inflammatory diseases because many biological settings do not conform to clean pathway boundaries. Instead, cells may activate overlapping caspases, inflammasome components, and necroptotic mediators in a temporally linked or compensatory manner.

In candidiasis, PANoptosis is an appealing conceptual model because *C. albicans* simultaneously imposes multiple forms of stress: pathogen sensing, hypha-associated membrane disruption, inflammatory cytokine exposure, oxidative imbalance, and metabolic pressure (Banoth et al., 2020). These converging signals make it plausible that mixed death phenotypes could arise, especially in severe infection or inflamed tissues where no single pathway fully explains the observed pathology. PANoptosis may therefore offer a language for describing crosstalk among death programs when pyroptosis, apoptosis-like signaling, and necroptosis-associated features coexist.

However, this framework should currently be used with caution in the Candida field. While mixed or overlapping death signatures are plausible and perhaps likely, definitive evidence for a bona fide PANoptotic signaling architecture in candidiasis remains limited. In many reports, multiple pathway markers are observed without establishing whether they are mechanistically integrated, sequentially activated, or simply reflective of heterogeneous cell populations undergoing different forms of death.

Thus, PANoptosis is best considered at present as a hypothesis-generating integrative concept rather than a settled mechanism in candidiasis. Its greatest value may lie in encouraging future studies to move beyond single-pathway interpretations and to examine how fungal virulence and host inflammation jointly configure combinatorial death responses.

### 4.6 Comparative interpretation of the current evidence

When the available literature is considered comparatively, a graded picture emerges. Pyroptosis is supported by the strongest mechanistic evidence and currently serves as the clearest pathway connecting *C. albicans* virulence to inflammatory host cell death. Apoptosis is relevant but more heterogeneous (Yang et al., 2025), often functioning as a background or context-dependent pathway rather than the primary explanatory mechanism. Necroptosis and ferroptosis are biologically plausible and increasingly discussed (Luo et al., 2025; Hou et al., 2026), but their roles remain more model-specific and less conclusively established. PANoptosis provides an attractive integrative framework, although emerging evidence from fungal infection models is beginning to provide mechanistic support for this integrated framework (Cai et al., 2025; Liang et al., 2025).

This hierarchy is important for both scientific interpretation and therapeutic translation. It argues against presenting all PCD pathways as equally established in candidiasis and instead supports a model in which pyroptosis forms the current mechanistic core, while other pathways expand the field’s understanding of tissue injury, pathway crosstalk, and disease heterogeneity.

## 5 Tissue-specific manifestations of programmed cell death in candidiasis

The same programmed cell death pathway may be protective in one anatomical context yet harmful in another. Candidiasis must therefore be understood through a tissue-specific lens, in which barrier structure, immune composition, and regenerative capacity collectively shape disease outcome. A central theme emerging from current research is that the pathological significance of programmed cell death in candidiasis cannot be generalized across tissues. The same fungal traits—hyphal growth, candidalysin production, cell wall remodeling, and metabolic adaptation—may trigger markedly different host outcomes depending on local barrier architecture, immune cell composition, inflammatory thresholds, and regenerative capacity. Consequently, cell death in candidiasis is best understood not solely as a molecular pathway problem, but also as a tissue-context problem.

This perspective helps explain why inflammatory death may be protective in one compartment yet drive symptomatic disease in another. At barrier surfaces, limited cell death can function as an alarm system that promotes fungal control and immune recruitment. However, if amplified beyond a tolerable threshold, the same response can compromise epithelial integrity, intensify leukocyte influx, and produce clinically significant inflammation. In deeper organs or disseminated infection, cell death may shift from a localized defense mechanism to a major determinant of organ dysfunction. The following sections therefore examine candidiasis by anatomical context, emphasizing how programmed cell death modifies the balance between antifungal defense and host injury.

### 5.1 Oral candidiasis: inflammatory defense at the cost of epithelial damage

Oral candidiasis provides one of the clearest examples of how programmed cell death simultaneously contributes to host protection and tissue pathology. The oral mucosa is continuously exposed to commensal and opportunistic fungi, requiring a response that is rapid enough to restrict invasive growth yet sufficiently controlled to preserve barrier function. When *C. albicans* shifts toward hyphal growth and produces candidalysin, epithelial cells are no longer passive surfaces but become active sensors and responders. They release cytokines, alarmins, and chemotactic factors, thereby recruiting myeloid cells and initiating a broader inflammatory program.

In this setting, inflammatory cell death—particularly pyroptosis-related responses—appears to contribute to early antifungal signaling. Such responses may help mobilize macrophages and neutrophils, enhance IL-1-associated inflammation, and strengthen local antifungal defense. However, the same mechanisms can also intensify epithelial disruption and symptomatic mucosal injury. Because the oral mucosa depends on intact epithelial architecture for both physical protection and immune regulation, excessive lytic cell death can quickly transform a protective alarm response into overt tissue pathology.

The importance of oral candidiasis in the broader PCD framework lies in its illustration of how barrier tissues tolerate only a narrow window of beneficial inflammation. Too little inflammatory signaling may permit uncontrolled fungal persistence, whereas excessive pyroptosis or oxidative injury may worsen barrier breakdown and prolong disease. This balance likely explains why oral candidiasis often reflects not only fungal virulence but also the host’s ability to spatially and temporally constrain inflammatory cell death.

### 5.2 Vulvovaginal candidiasis: immunopathology despite limited fungal invasion

Vulvovaginal candidiasis (VVC) highlights an important principle distinct from oral disease: severe symptoms may arise even when fungal invasion is relatively limited (Willems et al., 2020), and host pathology is driven disproportionately by inflammatory amplification. In the vaginal mucosa, *C. albicans* can trigger robust epithelial and neutrophil-associated responses, yet these responses do not always correlate with efficient fungal clearance (Ardizzoni et al., 2021). This partial uncoupling of inflammation from protection makes VVC a particularly relevant context for considering the pathological side of programmed cell death (Yano et al., 2018).

Although the precise contribution of individual PCD pathways in VVC remains incompletely defined, epithelial injury, neutrophil recruitment, and inflammatory mediator release create conditions under which lytic death responses may contribute to symptom generation (Yano et al., 2017; Roselletti et al., 2019). Here, the key issue may be less whether a single canonical pathway dominates and more whether the cumulative effect of tissue stress, leukocyte infiltration, and local damage exceeds the threshold for symptomatic inflammation. In other words, VVC may represent a setting in which the host response is sufficiently activated to produce tissue distress but insufficiently coordinated to ensure efficient fungal elimination.

This syndrome therefore underscores a broader conceptual point: in candidiasis, programmed cell death should not be evaluated solely by its antimicrobial intent. In some mucosal environments, death-associated inflammation may become clinically important even when its effect on fungal burden is modest. VVC is thus best understood as a model of Candida-associated immunopathology, where the costs of inflammation may be more evident than its protective benefits.

### 5.3 Gastrointestinal candidiasis and intestinal colonization: death control at a tolerogenic interface

The gastrointestinal tract presents a fundamentally different challenge because Candida often exists there as a commensal or low-level colonizer rather than an overt invasive pathogen. The host must therefore distinguish between tolerated presence and harmful overgrowth while preserving a highly specialized barrier densely populated by immune cells and microbial signals. In this environment, excessive inflammatory cell death could be particularly costly, as it may disrupt epithelial continuity, alter microbial homeostasis, and facilitate translocation of both fungi and bacteria.

Programmed cell death in the gut is therefore likely to be tightly regulated and deeply context-dependent. Limited death responses may help eliminate infected or damaged cells and maintain mucosal surveillance, but uncontrolled lytic death has the potential to destabilize intestinal barrier integrity and amplify inflammation beyond what is needed for fungal control. This issue becomes especially relevant in settings of antibiotic exposure, immunosuppression, inflammatory bowel disease, or critical illness, where colonization may shift toward pathogenic behavior and host tolerance mechanisms become less effective. Microbial contributions to the gut-liver axis are increasingly recognized in metabolic liver disease (Buchynskyi et al., 2025).

Compared with oral candidiasis, direct evidence for specific Candida-induced PCD pathways in the intestinal mucosa remains less defined. Nevertheless, the gut is conceptually important because it emphasizes that the principal danger may not always be overt fungal cytotoxicity, but rather the breakdown of a normally restrained host–microbe equilibrium. In this context, programmed cell death may function either as a homeostatic containment mechanism or as a trigger for barrier failure, depending on the surrounding inflammatory state.

### 5.4 Liver and deep-organ candidiasis: from local inflammatory death to organ dysfunction

In deeper tissues such as the liver, the significance of programmed cell death shifts again, and intestinal fungi can contribute to the development of alcoholic liver disease (Yang et al., 2017). Unlike mucosal surfaces, these organs are not designed for continuous microbial contact, and thus fungal invasion is more likely to provoke intensive myeloid activation, parenchymal stress, and collateral tissue injury. Here, cell death may no longer be confined to local barrier signaling but may instead become a direct driver of organ pathology. Importantly, direct invasive hepatic candidiasis should be clearly distinguished from the broader association between intestinal fungi and alcoholic liver disease, which may involve translocation-associated or microbiota-driven inflammatory effects rather than direct fungal invasion of the liver.

Hepatic and deep-organ candidiasis likely involve multiple interacting forms of host cell stress, including inflammatory cytokine exposure, oxidative injury, microvascular dysfunction, and immune-cell infiltration (Drummond et al., 2019; Wakade et al., 2026). Under these conditions, pyroptosis may contribute to inflammatory amplification, while apoptosis, necroptosis-like signaling, or ferroptosis-related lipid damage may become more relevant as secondary mechanisms of tissue injury. The general landscape of cell death in liver disease has been increasingly characterized (Stoess et al., 2024). The relative importance of each pathway remains incompletely resolved, but the overall pattern suggests that once fungal infection reaches deeper organs, the clinical consequence of cell death shifts toward loss of functional tissue integrity rather than localized mucosal defense alone.

This organ-level perspective is important because it broadens the implications of PCD beyond antifungal immunity. In deep candidiasis, the host may be harmed not only by fungal persistence but also by the cumulative burden of inflammatory and metabolic injury imposed on non-renewable or functionally critical tissues.

### 5.5 Disseminated and systemic candidiasis: when host defense becomes a driver of lethal pathology

Disseminated candidiasis represents the setting in which the protective–pathological duality of programmed cell death becomes most clinically consequential. Once *C. albicans* enters the bloodstream or multiple organs, host responses that are beneficial at localized sites may become amplified across a systemic inflammatory landscape (Liu et al., 2011; McFadden et al., 2025), and inflammatory ER stress responses can dictate the immunopathogenic progression of systemic candidiasis (Awasthi et al., 2023; Cheng et al., 2025). Myeloid activation, endothelial stress, cytokine release, oxidative injury, and organ-specific susceptibility can together transform regulated cell death from a compartmentalized defense mechanism into a contributor to multiorgan dysfunction (Lionakis et al., 2012; Zhao et al., 2025), with damage-associated molecular patterns (DAMPs) such as HMGB1 representing potential targets in sepsis (Rao et al., 2014). In the brain, pathogen-inducible antimicrobial neuropeptides such as PACAP can provide rapid and contextual host defense (Lee et al., 2021).

In systemic infection, pyroptosis remains a plausible central pathway because of its strong links to innate immune activation and cytokine maturation. However, the broader inflammatory and metabolic collapse associated with severe disease may also create conditions in which other death pathways become more visible (Weerasinghe et al., 2026), including apoptosis in immune or parenchymal compartments and ferroptosis-like injury associated with oxidative imbalance. In such settings, the interaction among pathways may matter more than any single mechanism in isolation (Allen et al., 2025).

The clinical relevance of this shift is substantial. A host response that is initially effective at recognizing and restricting fungal invasion may, if excessive or poorly resolved, contribute to vascular leak, tissue injury, and failure of critical organs. Disseminated candidiasis therefore illustrates the most extreme version of the framework proposed in this review: fungal virulence initiates host defense, but the resulting death-associated inflammation may itself become a major determinant of fatal outcome.

### 5.6 Comparative synthesis across tissues

Comparison across tissue compartments reveals several recurring principles. First, the impact of programmed cell death depends less on the nominal pathway alone than on the structural and immunological constraints of the affected organ. Barrier tissues such as the oral and vaginal mucosa can benefit from rapid inflammatory alarm responses, but they are also highly vulnerable to epithelial disruption. In the intestine, where tolerance and microbial coexistence are essential, even a moderate excess of lytic death may destabilize homeostasis. In deeper organs and systemic disease, cell death becomes increasingly linked to parenchymal injury and organ dysfunction.

Second, tissue-specific outcome reflects not only host architecture but also the degree to which inflammation remains coupled to fungal control (Liu et al., 2025). Oral candidiasis may involve a relatively functional defense response that becomes excessive, whereas vulvovaginal disease often illustrates a stronger dissociation between inflammatory symptoms and efficient fungal elimination. Disseminated candidiasis represents yet another state, in which the scale of host activation can outgrow local containment entirely.

Third, these differences argue against a universal therapeutic strategy aimed at suppressing all forms of cell death in candidiasis. The same intervention could be beneficial in one tissue and harmful in another. A more realistic framework is to identify which death pathways dominate in a given organ, at what stage of infection they become pathological, and whether modulating them would preserve antifungal defense or compromise it.

These tissue-level considerations provide the rationale for the next section, which focuses on translational implications and the opportunities and limitations of targeting programmed cell death in antifungal therapy.

The biological significance of programmed cell death in candidiasis varies across anatomical compartments. In oral and gastrointestinal mucosa, cell death responses are closely linked to epithelial defense, barrier maintenance, and IL-17-associated immunity. In vulvovaginal candidiasis, inflammatory cell death may contribute disproportionately to symptoms relative to fungal burden. In liver and deep-organ settings, direct invasive infection should be distinguished from translocation-associated inflammatory effects. In disseminated candidiasis, early inflammatory death responses may support fungal control but can later contribute to systemic immunopathology and organ dysfunction. Thus, the same programmed cell death pathway may be beneficial or harmful depending on tissue context, infection stage, and host response architecture.

## 6 Therapeutic implications of targeting programmed cell death in candidiasis

The therapeutic relevance of programmed cell death in candidiasis lies precisely in this balance between protection and pathology. A host death pathway becomes a meaningful target not simply because it is activated, but because its contribution to tissue injury can be separated—at least in principle—from its role in antifungal defense. The growing recognition of programmed cell death (PCD) in candidiasis raises an important translational question: can modulation of host death pathways improve outcomes beyond conventional antifungal therapy? In principle, the answer is yes. Because PCD contributes not only to fungal containment but also to immunopathology, tissue damage, and possibly organ failure, selective intervention in host death pathways may help rebalance the host response. However, this therapeutic concept is inherently complex. The same pathway that drives collateral injury may also be required for early antifungal defense. As a result, the goal should not be indiscriminate suppression of cell death, but context-specific modulation of pathological death responses while preserving protective immunity. We have summarized the information in **Figure 2**.

**Figure 2:**
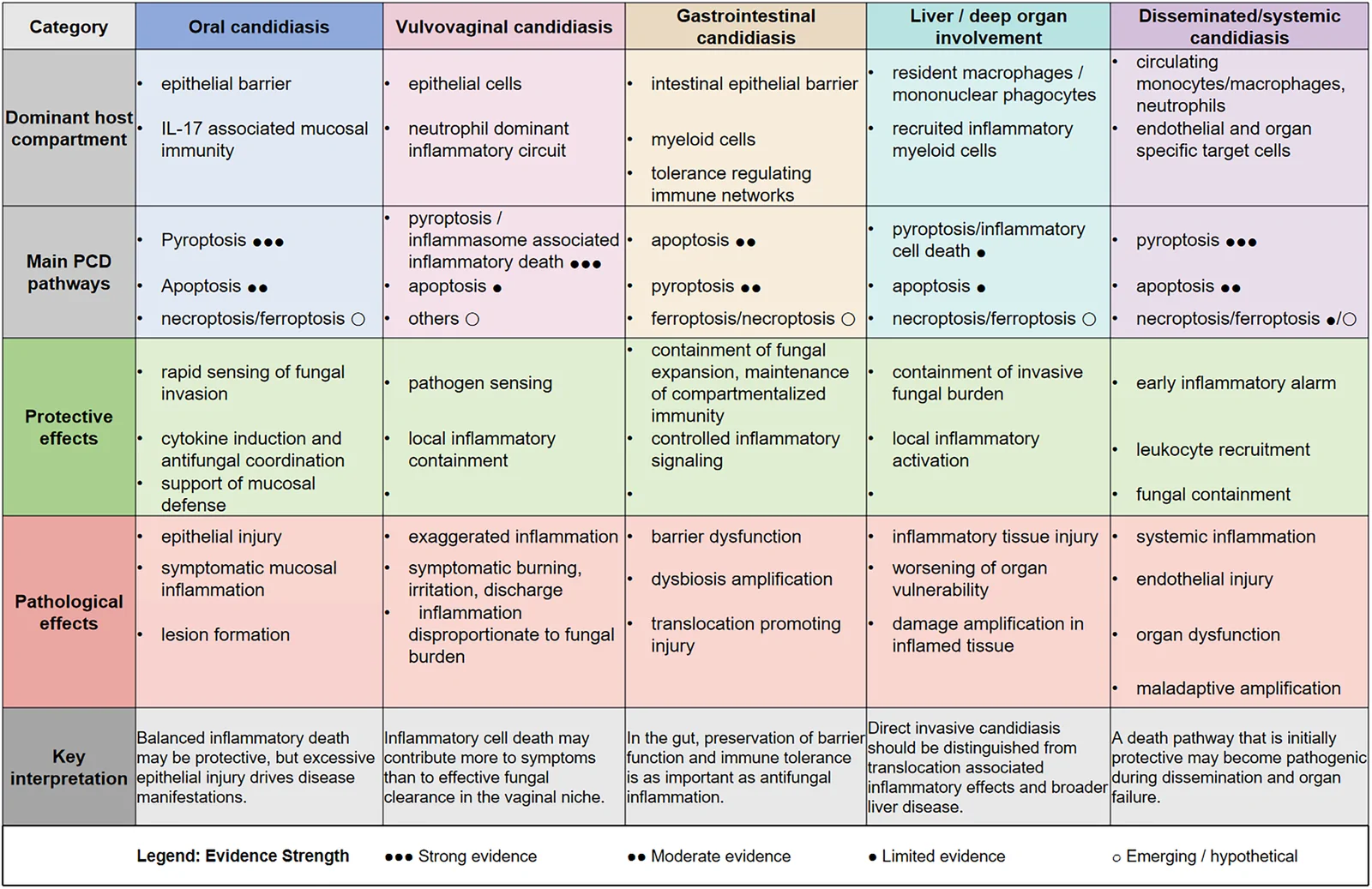
Tissue-specific manifestations and consequences of programmed cell death in candidiasis.

### 6.1 Pyroptosis as the most actionable—but also most delicate—therapeutic target

Among the currently discussed pathways, pyroptosis is the most immediately actionable candidate for host-directed intervention because it is the best-defined mechanistically and the most clearly linked to inflammatory amplification in *C. albicans* infection. Targeting inflammasome activation, caspase-1 signaling, gasdermin-mediated membrane permeabilization, or downstream IL-1-family responses could, in theory, reduce excessive tissue injury in settings where inflammatory pathology outweighs antifungal benefit (Gander-Bui et al., 2023). NLRP3 has also been proposed as a therapeutic target in alcoholic liver disease, illustrating the broader translational interest in inflammasome modulation (Brahadeeswaran et al., 2023).

Yet pyroptosis is also the pathway for which therapeutic overcorrection may carry the greatest risk. Inflammasome-dependent signaling contributes to leukocyte recruitment, cytokine maturation, and the orchestration of antifungal immunity. Broad inhibition could therefore blunt fungal control, delay immune activation, or alter downstream adaptive responses. This is especially relevant early in infection or at barrier sites, where rapid inflammatory signaling may be necessary to prevent fungal persistence or invasion (Fang et al., 2025).

For these reasons, pyroptosis should be viewed as a tunable target rather than a pathway to abolish. The most promising strategies may involve partial, time-restricted, or tissue-specific modulation rather than complete blockade. Combination approaches may be particularly attractive—for example, pairing antifungal agents with selective anti-inflammatory interventions in patients whose disease is dominated by immunopathology rather than uncontrolled fungal growth. The major translational challenge is therefore not whether pyroptosis matters, but how to identify the clinical settings in which its inhibition would reduce damage without undermining host defense.

### 6.2 Apoptosis, necroptosis, and ferroptosis: emerging host-directed opportunities with different levels of readiness

Compared with pyroptosis, other PCD pathways are less mature as therapeutic targets in candidiasis, but they may still offer important opportunities. Apoptosis is conceptually relevant because excessive loss of epithelial, immune, or parenchymal cells may compromise barrier function and tissue repair. However, its broader physiological role in homeostasis makes non-selective anti-apoptotic intervention difficult to justify unless a specific pathological context can be identified.

Necroptosis, if validated more firmly in fungal disease, could become an attractive target in situations of severe inflammatory injury because of its membrane-disruptive and pro-inflammatory nature. Pharmacologic inhibition of necroptosis-related signaling has shown value in other inflammatory conditions, but in candidiasis the current evidence remains insufficient to support strong translational claims. At present, necroptosis should be considered a candidate pathway for future therapeutic exploration, not a clinically mature target.

Ferroptosis may represent a distinct type of opportunity because it links cell death to iron metabolism, lipid peroxidation, and redox imbalance—processes increasingly recognized as contributors to inflammatory tissue damage. If ferroptosis is confirmed as a significant component of organ injury in selected forms of candidiasis, then antioxidant or lipid-protective strategies may have translational potential, as has been explored for blood cell-related diseases (Chen et al., 2022). This possibility is especially interesting because ferroptosis-oriented interventions might attenuate metabolic tissue injury without directly suppressing all antimicrobial inflammatory signaling. Nonetheless, the field is still at an early stage, and stronger mechanistic evidence is needed before ferroptosis can be placed alongside pyroptosis as a realistic host-directed target.

### 6.3 Why combination and precision approaches are more realistic than pathway-wide inhibition

A major lesson from current evidence is that successful intervention in candidiasis is unlikely to come from broad inhibition of a single cell death pathway across all patients and disease settings. Instead, the biology of candidiasis argues for precision-oriented, combination-based strategies. Antifungal drugs reduce pathogen burden, whereas host-directed modulation of PCD may reduce the inflammatory and metabolic damage that persists despite pathogen control. These two goals are complementary rather than interchangeable. For example, pharmacological blockade of CCR1 protects mice from systemic candidiasis of hematogenous origin, supporting the feasibility of host-directed immunomodulation in selected systemic settings (Lionakis et al., 2017).

Such an approach is especially relevant because fungal burden and host inflammatory injury do not always correlate tightly. Some patients or models may be dominated by insufficient fungal control, while others may exhibit disproportionate immunopathology relative to organism load. In the latter context, adjunctive modulation of cell death-associated inflammation may improve tissue outcomes even if it has little direct antifungal effect. Conversely, in patients with poorly controlled fungal proliferation, suppressing inflammatory death too early or too broadly may be counterproductive.

This logic supports the development of stratified treatment frameworks that incorporate tissue site, disease severity, inflammatory phenotype, and possibly molecular biomarkers of death pathway activation. Ultimately, the translational value of PCD biology in candidiasis will depend on whether these pathways can be used not merely as mechanistic explanations, but as guides for patient-specific therapeutic decisions.

### 6.4 Key barriers to clinical translation

Despite growing interest, several major barriers currently limit the therapeutic exploitation of programmed cell death in candidiasis. First, most mechanistic data derive from *in vitro* systems or selected animal models, and the extent to which these findings predict human disease remains uncertain. Second, the same molecular marker may reflect different biological processes in different tissues or cell populations, complicating efforts to assign pathway dominance *in vivo*. Third, candidiasis is clinically heterogeneous: superficial mucosal disease, deep-organ infection, and disseminated candidemia are unlikely to respond similarly to the same host-directed intervention.

A further challenge is that cell death pathways are dynamic rather than static. A pathway that is protective early in infection may become pathological later, and compensatory switching among death programs may occur when one pathway is blocked. These features make therapeutic timing and patient selection central concerns. Thus, before host-directed targeting of PCD can be realistically advanced, the field requires better biomarkers, more tissue-resolved mechanistic studies, and clearer distinctions between protective and maladaptive death responses in defined clinical syndromes.

At present, however, the field is not yet ready for broad clinical application of cell death-targeted therapies in candidiasis. This limitation points to a broader need: a more precise and integrated framework that can define pathway dominance, tissue specificity, and therapeutic windows with greater biological and clinical clarity.

## 7 Future perspectives and conclusions

Looking forward, the central task is not merely to catalogue additional death pathways, but to refine how programmed cell death is conceptualized in fungal disease. Future progress will depend on integrating molecular mechanisms with tissue context, infection stage, and host heterogeneity. Current evidence supports a more integrated view of candidiasis in which fungal virulence, host sensing, and tissue-specific immunity converge on programmed cell death as a major determinant of outcome. Importantly, these death pathways should not be interpreted as isolated modules of equal significance. Rather, the available literature supports a hierarchical and context-dependent framework. Pyroptosis is the most strongly established pathway and presently serves as the clearest mechanistic bridge between *C. albicans* virulence and inflammatory host injury. Apoptosis remains relevant but comparatively heterogeneous, while necroptosis and ferroptosis represent promising yet still emerging dimensions of fungal immunopathology. PANoptosis offers a useful integrative concept for overlapping death responses, but in candidiasis it remains more interpretive than definitive.

A major challenge for the field is now to move beyond pathway labeling toward spatiotemporal and cell-type-resolved understanding. Which cells die, when they die, and in which tissue context may be more important than whether a single molecular marker is present. Future studies should therefore prioritize approaches capable of resolving heterogeneity across infected organs and immune compartments, including single-cell profiling, spatial transcriptomics, advanced imaging, and genetically precise pathway interrogation in tissue-specific models. These strategies will be essential for distinguishing true pathway activation from secondary injury, mixed phenotypes, or population-level averaging.

Another important direction is to better define the relationship between fungal virulence programs and host death decisions. Hyphal morphogenesis, candidalysin production, metabolic adaptation, and cell wall remodeling are often discussed separately, yet *in vivo* they likely operate as convergent inputs that shape the threshold, intensity, and type of host cell death. Understanding this interface may clarify why some forms of candidiasis remain localized and self-limited, whereas others progress toward destructive inflammation or organ dysfunction.

The field should also remain open to emerging or less-defined PCD frameworks, but with appropriate caution. PANoptosis may prove useful for explaining mixed inflammatory death phenotypes in severe candidiasis, especially where pyroptotic, apoptotic, and necroptotic features overlap. By contrast, pathways such as cuproptosis are currently better viewed as speculative or exploratory concepts in the context of candidiasis. Although fungal infection clearly perturbs host metabolism and micronutrient handling, there is not yet sufficient evidence to place cuproptosis among the established or even leading candidate mechanisms of host cell death in Candida disease. Its greatest value at present lies in reminding the field that metabolic forms of injury may deserve broader investigation.

From a translational perspective, the most promising future direction is not the blanket suppression of host cell death, but the development of precision host-directed therapies that account for tissue context, pathway dominance, and stage of infection. Such strategies will likely require integration of antifungal treatment with selective modulation of harmful inflammatory or metabolic death responses. Whether this goal can be achieved will depend on improved biomarkers, more faithful disease models, and a clearer separation between protective immunity and pathological self-injury.

In conclusion, programmed cell death is not a peripheral consequence of candidiasis but a central arena in which fungal invasion and host defense are negotiated. The outcome of this interaction is neither uniformly protective nor uniformly harmful. Instead, candidiasis is shaped by how fungal virulence factors engage host sensing pathways, how those signals are amplified within specific tissues, and how the resulting death programs are balanced between containment and damage. Recognizing this complexity provides a more coherent framework for interpreting existing evidence and a more realistic foundation for future therapeutic innovation.
